# The development of SIBS-ONLINE, a group-based video conference intervention for siblings of children with chronic disorders and their parents

**DOI:** 10.1016/j.pecinn.2023.100220

**Published:** 2023-09-22

**Authors:** Torun M. Vatne, Yngvild B. Haukeland, Krister W. Fjermestad

**Affiliations:** a Frambu resource centre for rare disorders, Sandbakkvn 18, N-1404 Siggerud, Norway; b Adults for children, Lille Grensen 5 0159, Oslo, Norway; c Department of Psychology, University of Oslo, Po box 1094, N-0317, Oslo, Norway

**Keywords:** Chronic childhood disorders, Siblings, Group intervention, E-health, Video conference

## Abstract

**Objective:**

During the last decade a knowledge-based group intervention called SIBS, aiming to prevent psychological difficulties in siblings of children with chronic disorders, has been developed and empirically evaluated. The results are promising, but SIBS has been difficult to implement in areas with low population density. To address the needs of low-density health regions a video conference-based version of SIBS, “SIBS-ONLINE”, was developed.

**Method:**

SIBS-ONLINE was developed in three phases: 1) Gaining experience by delivering five support groups for siblings during the Covid-19 pandemic over video conference; 2) Developing the SIBS-ONLINE intervention based on the experiences of Phase 1; 3) Pilot testing the SIBS-ONLINE intervention with four groups (14 families).

**Results:**

The end-product was SIBS-ONLINE, an intervention for siblings aged 10–16 years and their parents. SIBS-ONLINE consists of three separate sibling and parent groups and three joint sessions delivered over video conference. Intervention materials to enable delivery were developed, such as a manual comprising specification of communication techniques in video conference groups, technical advice, and a detailed description of sessions.

**Conclusion:**

The developmental process resulted in a feasible group-based video conference intervention for families of children with chronic health disorders.

**Innovation:**

A unique group-based video conference intervention is described and general advice for developers and deliverers of future interventions provided.

## Highlights

• Video conference groups meet needs of families of children with chronic disorders.
• SIBS-ONLINE is a preventive video conference intervention for siblings.
• SIBS-ONLINE is feasible and acceptable.

## 1 Background

Sibling relations, our longest lasting social bonds, are important for childhood development. There is considerable documentation that growing up with a brother or sister with a chronic disorder represents risk for typically developing siblings (herein, siblings) [1]. Chronic disorders are physical or mental health conditions, diseases, and/or disabilities with long duration, affecting multiple domains of a child's physical and/or intellectual functioning. Review studies and meta-analyses have shown that the risks for siblings entail poorer mental health (e.g., depression, anxiety, behavior problems, negative self attributes) and poorer family communication (i.e., less warm and open communication) than for controls [[1], [2], [3], [4], [5]].

We estimate that over a hundred thousand children in Norway are siblings of a child with a chronic disorder [6,7]. Psychological support for these siblings is required by Norwegian law; health personnel are imposed to safeguard the need for information and necessary follow-up of siblings [8] and municipalities are responsible for acting to prevent psychological maladjustment in high-risk child populations [9,10]. However, it does not seem like health providers are able to act on this responsibility. A 2019 survey in which 59% of Norwegian municipalities participated, showed that the support offered to siblings is scarce and that evidence-based interventions are lacking [11].

The team behind the current paper has developed a manual-based 5-session preventive group intervention for siblings and parents of children with chronic disorders called SIBS (the name SIBS is short for sibling) [12]. The aim of SIBS is to strengthen parent-child communication, and thereby improve psychological adjustment and diagnostic knowledge in siblings. In an open trial with 99 families, we found significantly improved sibling mental health, disorder knowledge, and family communication from baseline to 3- and 6-month follow-up, with small to medium effect sizes (*d* = 0.22 to 0.64) [13]. The SIBS research team are currently conducting a randomized controlled trial of the effect of SIBS with 288 families [14].

Implementation studies have shown high participant satisfaction with SIBS and high manual adherence by SIBS group leaders [13,15]. However, difficulties with recruiting families and few resources in the municipalities are reported as implementation barriers [11]. In Norway, many families live in remote areas which implies long traveling distances to service sites. This may inhibit families from participating in SIBS or other family services that require physical attendance. In addition, many Norwegian municipalities have small populations, and thus, forming homogenous groups in terms of sibling age and child disorder type is difficult. Small municipalities also reported to lack the personnel to be able to deliver SIBS in person [11].

The recent decades have seen a fast development in the field of e-health. Video conferencing is one way of delivering e-health interventions. Psychological interventions for children delivered using video conferencing are described as potentially cost effective, convenient, and available to a more diverse population than in person interventions [16]. Group-based video conference interventions for adults have been found to be feasible, acceptable, effective and reliable, but IT support, communication strategies and group leader training is important for feasibility [17]. Children generally have positive attitudes towards e-health interventions as it is reported to reduce stigmatization and barriers for seeking help and increase feelings of connectedness and accessibility of help [18]. However, although support group interventions for children delivered using video conferencing is described as an emerging field [16], we have not been able to identify research on such interventions.

The group behind this study were experienced with web-based counseling with parents as this is one of the services provided at Frambu resource centre for rare disorders [19] and has a lot of experience with conducting in-person child and parent groups. To meet the psychosocial needs of the sibling population within the frames of Norwegian geography and the resources of the municipal health care system, we decided to further develop and adapt the SIBS intervention so that the intervention could be offered over video conference. This paper describes the process of developing this intervention called “SIBS-ONLINE” while identifying and managing factors that may affect the feasibility, acceptability, effectiveness, and implementation of the intervention. The paper aims to provide clinicians and researchers with inspiration and knowledge about how to digitalize interventions originally developed for a physical in-person format.

### 1.1 The SIBS intervention

The original face-to-face SIBS intervention is an integrated parent-sibling intervention consisting of three child group sessions, three parent group sessions and two joint sessions. The intervention can be delivered over two days or sessions spread over days or weeks as desired by the deliverer. The intervention was originally developed for siblings aged 8–16 years, and siblings join in age matched groups of 3–7 children with a maximum of three years age span. It is recommended that siblings in one group represent disorders with similar features, but this is not required. The SIBS sessions cover three topics: Getting familiar in the group and the intervention, the diagnosis of the affected child and emotional experiences of siblings. Table 1 describes the sessions in SIBS, in the order they are delivered, their aim and content. Child and parent session 1, 2 and 4 may be delivered at the same time or the parent sessions after the child session with the same number. The target group, content and form of SIBS is based on a thorough developmental process involving pilot testing with 68 families [12].

**Table 1:** Description of sessions, aims and content of the SIBS intervention.

Topic	Session	Time	Aim	Content
Information and relation building	1 Child	20 min	Get to know each other. Get information about the intervention.	Games to get to know each other. Information about SIBS. Making rules.
	1 Parents	20 min	Get to know each other. Get information about the intervention.	Games/tasks to get to know each other. Information about SIBS.
The diagnosis	2 Child	60 min	That children become aware of own information need and formulate questions for parents.	Exploration of name, symptoms, cause, treatment consequences and timeline of the diagnosis using e.g. body map and drawings. Formulating a joint and individual questions for parents.
	2 Parents	60 min	That parents become aware of the value of talking about the diagnosis with siblings and get to know and practice communication techniques.	Psychoeducation (standardized film). Examples of communication for discussion (standardized films). Role play and practice communication based on joint question from sibling group. Prepare for joint session.
	3 Joint	Min 20 Max 60	That children and parents talk about the diagnosis and parents practice techniques with supervision.	Siblings pose individual questions as starting point for conversation. Parents practice techniques. Group leader provides supervision.
Emotional experiences	4 Child	60 min	That children become aware of their emotional/supportive needs and formulate a wish for parents.	Introducing emotions using e.g. emotion cards, mime game. Explore sibling related experiences. Formulate a joint and individual statements of “what I wish were different” for parents.
	4 Parents	60 min	That parents become aware of the value of talking with children about emotional experiences as siblings and get to know and practice communication techniques.	Psychoeducation (standardized film). Examples of communication for discussion (standardized films). Role play and practice communication based on joint statement of “wish” delivered from sibling group. Prepare for joint session.
	5 Joint	Min 20 Max 60	That children and parents talk about emotional experiences and parents practice techniques with supervision.	Siblings express their individual “wish” as starting point for conversation. Parents practice techniques. Group leader provides supervision. Parent and sibling discuss solutions related to the “wish”. Parents and sibling sign agreement about communication at home.

The complete SIBS material comprises a detailed manual describing the content and the implementation of SIBS for group leaders, example movies of parent-child dialogues, and psycho-educative movies focusing on understanding siblings' information and emotional needs and how to enhance communication about these needs for use in parent sessions, a parent folder containing the information from the psycho-educative movies, and a sibling folder containing tasks for the child sessions. In addition, there is a standardized course package for group leaders to become licensed SIBS group leaders. The training comprises a 45-min online course, a two-day practical course, and a voluntary semi-annal webinar. For more information about the course package see www.sibs.no.

SIBS is led by four clinicians, such as school nurses, family therapists or psychologists. The child group has one group leader, who is responsible for complying with the manual and leading the conversation. A facilitator looks for and ensures that all children are involved, their needs seen and understanding ensured as well as aids the group leader in keeping track of time. The parent group is also led by two clinicians with similar roles as in the child group. If parent sessions are run after child sessions SIBS might be led with only two clinicians in total.

## 2 Methods

The video conference-based version of SIBS, “SIBS-ONLINE”, was developed in three phases. Phase 1 involved gaining experience with delivering a group-based video conference intervention for children. Phase 2 involved revision of the original face-to-face SIBS intervention into the SIBS-ONLINE intervention based on the experiences from Phase 1. Phase 3 involved pilot testing of the SIBS-ONLINE intervention and final revisions based on the experiences, including participant and group leader feedback. In the developmental process the definitions of feasibility, acceptability effectiveness, and implementation as formulated by Banbury and colleagues [17] were applied. Feasibility thus concerns possible technology factors that facilitate or inhibit connections of groups of people and enable facilitation and discussion. Acceptability concerns the extent to which the intervention is suitable, satisfying, or attractive to participants. Effectiveness relates to the extent to which the intervention has an effect upon participants health outcomes, that is changes something in the person. Implementation concerns the extent to which the intervention can be successfully and reliably delivered to participants. The methods of each of these three phases are presented separately below.

### 2.1 Methods phase 1

#### 2.1.1 Sample and procedures phase 1

To gain experience with delivering a group-based video conference intervention for children, we offered support groups for siblings during the initial stage of the Covid-19 pandemic in 2020. Norwegian children were home schooled for a long period in 2020, and their interaction with peers was strictly limited due to the pandemic. Due to the fragile medical status of many children with chronic disorders, many siblings were socially isolated for several months. In addition, medical services for families were limited during the pandemic, leaving parents with an increased caregiving burden. Our needs for gaining experience with online groups thus coincided with an increased need of video conference-delivered psychosocial support for siblings. We used an open invitation approach, with no strict inclusion criteria except age (10 to 16 years) and having a brother or sister with a chronic disorder. In Phase 1, the lower age limit was set at 10 years as younger children, based on our experience with in-person groups, need closer follow-up than is possible to provide online. We advertised the groups at the web and Facebook pages of Frambu resource centre for rare disorders, regional habilitation services across Norway, and various patient organizations (non-profit patient focused organizations whereby patients and/or caregivers represent a majority of members in governing bodies) such as The Norwegian Autism Association and The Norwegian Association for Children with Congenital Heart Disease. Interested participants emailed the project coordinator who scheduled groups and organized participants in groups of maximum four siblings with a maximum 3-year age range. We aimed to recruit minimum 3, maximum 7 siblings for each group.

All participants were registered with age, mobile phone number, and/or email address for at least one parent. The communication platform applied for the groups was Norwegian Health Net (nhn.no), a secure licensed system for video conferencing owned and run by the Norwegian Health Department. The health provider pays for the license, but the use is free for families. The participants log on to join.nhn.no, dial a unique conference number, and provide a password to enter the online group. The screen image comprises small images of each participant, and the image of the currently speaking participant expands during the time (s)he speaks. The system can be joined via computers, tablets, and smart phones.

The participants were informed that the groups were conducted as a first try of delivering a group-based video conference intervention for siblings and that their feedback was voluntary and would be used in developing online interventions. The data collected did not include identifiable or clinical data concerning the participants. Therefore, we did not need approval from the local institutional review board.

#### 2.1.2 Program description phase 1

The participants were offered 3 × 60-min sessions, conducted weekly for 3 weeks. A simple and flexible manual for the program was developed by the SIBS developers. The groups were similar to the original SIBS intervention in terms of the number of participants, group leadership styles and use of communication techniques. However, the sessions content differed from the original SIBS as these groups were focused on experiences related to the ongoing pandemic. Session 1 comprised provision of the rationale for the groups (support during the pandemic), creating group rules, warm-up exercises, and a presentation round of each participant and their family. Next, everyday life experiences of the participants related to the pandemic were discussed ending up with a round of coping advice presented by each participant. Session 2 started with a brief recap of the previous session and a repetition of group rules. Next, each participant's daily routines and change in mood during the last week were reviewed and discussed. Parental functioning and social support were addressed. The session ended with a round on advice on how to enhance mood and enhance access to social support. Session 3 was a recap and repetition session in which we addressed remaining issues from previous sessions.

#### 2.1.3 Measures and analyses phase 1

We sent an anonymous online questionnaire developed for this stage of the study to the participants about their experience of the feasibility, acceptability, and effectiveness of the intervention, in total 22 questions. We present quantitative data from these feedback forms in the results section. In addition, challenges in relation to feasibility, acceptability, effectiveness, and implementation and was discussed by group leaders between each group session. The challenges and suggestions on how these could be solved in SIBS-ONLINE were noted.

### 2.2 Methods phase 2

We used the results from the participant feedback forms and the notes from discussions with group leaders as a basis for the work with developing a preliminary version the SIBS-ONLINE intervention. This was done by reviewing the original SIBS intervention in light of the feedback and results of Phase 1, making changes that addressed the aspects reported to affect feasibility, acceptability, effectiveness, and implementation of video conference groups. To guide the group leaders in Phase 3, a preliminary pilot version SIBS-ONLINE manual was developed.

### 2.3 Methods phase 3

#### 2.3.1 Sample and procedures phase 3

To pilot test the preliminary version of SIBS-ONLINE intervention manual, we invited siblings and one of their parents to join in groups throughout 2021. We used an open invitation approach similar to that of Phase 1, with no strict inclusion criteria except age 10–16 years and having a sibling with a chronic disorder. We advertised the groups on the webpages and Facebook pages of Frambu resource centre for rare disorders, local habilitation services across the country, and various patient organizations.

All participants were registered with age, mobile phone number, and/or email address for at least one parent. The contact information was made available for group leaders during the groups (informed by Phase 1) to enable provision of technical support by phone and closer follow up of individuals if needed during sessions. The communication platform applied for the groups was Zoom (zoom.us), as this platform provides high quality picture of all participants, enables creations of multiple breakout rooms, and has a chat function (need informed by Phase 1, in which we used a different platform join.nhn.no). The participants were provided with the opportunity to test Zoom guided by a project group member before session 1 of the intervention. Shortly before each group session participants were sent a link by email to enter the group online. The Zoom meeting was locked when all participants had joined to ensure confidentiality. The system can be joined via computers, tablets, and smart phones.

#### 2.3.2 Measures phase 3

The participants were informed that the groups were conducted as a first try of delivering the intervention “SIBS-ONLINE” and that their feedback were voluntary, appreciated, and would inform further development of the interventions. Participant experiences of acceptability and experienced outcomes (proxy for effectiveness) of SIBS-ONLINE were gathered verbally in the evaluation part at the end of session 5, written down by group leaders, and sent to the developers. Group leader experiences of the feasibility, acceptability, outcomes (effectiveness), and implementation of SIBS-ONLINE were gathered by email after each session. The current report does not include identifiable or clinical data concerning the participants. Therefore, we did not need approval from the local institutional review board.

## 3 Results

### 3.1 Results phase 1

Fifteen siblings aged 10 to 16 years (14% boys; 86% girls) participated in 5 groups in Phase 1. These were siblings of children with various chronic disorders including rare genetic disorders and autism spectrum disorders. The group leaders were clinical psychologists with 5 to 17 years of clinical experience, all involved in developing the original SIBS [12], and all with considerable experience with running in-person sibling groups.

In terms of feasibility, 78% of the participants in the groups had never attended an online group before, the rest had attended school-based online groups. The majority (80%) joined via laptop, two by stationary computers, and one by a smartphone. Just over half (55%) reported minor to moderate problems with sound or image. All (100%) reported to have felt at least somewhat confident with the technical aspects, and when asked to compare their technical insights to those of their parents 67% reported to be equally or more confident. For a summary of group leaders' experience of feasibility, more specifically technical challenges during sessions, and suggestions on how these could be solved in SIBS-ONLINE, see Table 2, columns a and b.

**Table 2:** Group‑leader experience of challenges to feasibility, solutions and implication for development of SIBS-ONLINE.

a) Stage	b) Challenge	c) Solution	d) Implication⁎
Preparation	Participants lack computer or have difficulties logging on	Allow participation by smartphone, tablet or computer	Manual
	Poor timing of session school/−workwise	Allow for flexible delivery of in terms of time of the day	Pilot
	Group leader network is unstable	Prepare providers for both roles to enable facilitator to continue the session	Training
	Participants are unfamiliar with online system	Provide a test of the platform prior to group session 1	Pilot
		Provide information in session1	Manual
Sessions	Unstable devices (sound and movement)	Inform about placement of device in session 1	Manual
	Young participants fiddling and moving around	Further limitation of age range	Research
		Make rules in session 1	Manual
	Poor sound	Repeat participant statements	Manual
	Participants loses sound or picture	Have phone numbers available for assistance by facilitator	Manual
	Picture mode causes poor image of participants	Use an online platform with better visual quality	Manual

⁎ Manual = incorporated in SIBS-ONLINE manual draft, Pilot = incorporated in planning of the pilot groups, Training = incorporated in SIBS-ONLINE group leader training, Research = topic for future research.

In terms of acceptability, the participants reported that the topics discussed in the group were “important” or “very important” and that no important topics had been left out. The majority (93%) of the participants reported that privacy was respected during sessions, but one reported to have been disturbed by a family member. All participants reported that the group leader and the other participants had listened to them “much” or “very much”, on separate questions. All experienced that the group leader had been equally responsive across the participants.

In terms of effectiveness, all reported at least “*adequate*” support from the group leader and at least “*much*” support from other participants. The majority (78%) reported they felt they could support other participants. Most participants (78%) reported they had received “*good*” or “*very good*” advice from other participants. In terms of experienced outcomes (proxy of effectiveness), 56% reported that taking part had made coping with their daily life at home COVID-19 pandemic at least somewhat easier.

To an open-ended question about the most positive aspects of the groups, most comments concerned meeting others in the same situation. When reported, the most negative aspects of the groups concerned technical problems. One participant mentioned tiredness after the school day as interfering with the group, and one mentioned it may have been better to meet in real life.

For a summary of the group leaders' experienced challenges in relation to acceptability, effectiveness, implementation, and suggested solutions, see Table 3, columns a-c.

**Table 3:** Group‑leader experience of challenges to acceptability, effectiveness, and implementation in phase 1 and solutions and implications for development of SIBS-ONLINE.

a) Aspect	b) Challenge	c) Solution	d) Implication⁎
Acceptability	Participants/GL's talk at the same time	Structure turn taking	Manual
		Address participants by name	Manual
		Ask them to raise hands	Manual
		Facilitator should mainly use non-verbal facilitating responses	Manual
	Lack of responses	Provide time	Manual
		Ask for response using names, but allowing to say “pass” Look for nonverbal responses and address them	Manual Manual
Effectiveness	Lack of pro-social responses in group	Look for, and highlight, nonverbal signals form participants	Manual
Implementation	Awkward time waiting for participants to log on	Mute sound and picture while waiting	Pilot
		Repeat “we are waiting for X and will start in a few minutes”	Pilot
	Physical”Warm up” activities not possible online	Include new activities more suited for online platforms	Manual
	Participant shares concerning information	Schedule an individual meeting after the group session	Manual
		Contact parents after the session	Manual

⁎ Manual = incorporated in manual draft, Pilot = incorporated in planning of the pilot group.

### 3.2 Results phase 2

The result from Phase 2 was a preliminary version of SIBS-ONLINE with a corresponding draft of the SIBS-ONLINE manual to guide the group‑leaders in Phase 3. Aspects from Phase 1 incorporated in the manual draft, planning of the pilot groups, and training of group leaders are described in Table 2 column c and Table 3 column d.

### 3.3 Results phase 3

Fourteen siblings aged 10 to 15 years (36% boys, 64% girls) and one of their parents participated in four groups in Phase 3. These siblings had brothers or sisters with various chronic disorders including rare genetic disorders and autism spectrum disorders. The group leaders were three clinical psychologists, four psychology students, and an education specialist. The three clinical psychologists were the developers of the original SIBS with considerable experience running sibling groups. The remaining group leaders were all licensed SIBS group leaders who had completed an online course and a two-day practical course in SIBS and had observed the online support groups run in Phase 1 for learning purposes.

Siblings and parents provided verbal feedback about the experienced acceptability and effectiveness of SIBS-ONLINE. Their positive experiences, suggested areas for improvements, and implications for revision of SIBS-ONLINE and the corresponding intervention material can be found in Table 4. The group leaders expressed challenges in relation to feasibility, acceptability, effectiveness, and implementation. These challenges, the group leaders' suggestions for improvement, and the solutions included in the revised SIBS-ONLINE intervention and the corresponding intervention material are described in Table 5.

**Table 4:** Participant feedback of acceptability and effectiveness SIBS-ONLINE and implications for revision of SIBS-ONLINE.

	Positive experience	Area of improvement	Implication
Child groups	Nice to meet others (c)	Involve all children (c)	Take rounds. Facilitator follows up on questions
		Not meeting participant expectations of social meetings with likeminded (c)(p)	Provide more information about the format and the aims of SIBS-ONLINE before and during the intervention
	Easier to talk about difficult things when at home (c)		
Parent groups	Online groups make participating easier (p)	Time of the day is important for participation (p)	Place parent sessions after working hours
	Good with examples on how to talk with children (p)	Advanced language. (p)	Translate the SIBS target behaviors into more common language
		Make the films and examples more diverse in terms of age (p)	Both older and younger figures included in the revisions of the information and psychoeducation films
		A wish for more time to share experiences with other parents (p)	Provide more information about the format and the aims of SIBS-ONLINE before and during the intervention.
Joint sessions	God to get the opportunity to talk (p)(c)	Did not get answers to my questions (c)	Be thorough with explaining that parents are instructed to postpone answering questions in child session 2

*c = expressed by children, p = expressed by parents.

**Table 5:** Group leader experiences of challenges related to feasibility, acceptability, effectiveness and implementation, suggested solutions and suggestions included in the revision of SIBS-ONLINE.

Session	Challenge	Suggestion	Solutions in revision of SIBS-ONLINE
1 children	Children being uncertain of what to do when technical errors (F)	Include information about Zoom and support in session 1	As suggested
1 parents	Information provided about SIBS-ONLINE dependent on group leader competence (I)	Make an information film to replace verbal information	As suggested
2 children	Problems finding a good alternative to drawing body-maps on a flip over to explore the diagnosis (I)	Find a program that can easily be used to draw in Zoom	A document with body map to share and draw on developed. Tips on programs to use included in the manual
	Sending topics for discussion with parents by chat feels unsecure⁎ (I)	Thoroughly inform participants on how to send private chat	Lock participant chat to only private chats to group leaders⁎
2 and 4 parents	Psychoeducation section too long and the message unclear (A)	Revise manuscript and make a shorter film	New 5- min animated psycho-educative film developed
	Uncertainty of how to use the common question/challenge for discussion (I)	Make parents provide word by word descriptions possible responses	Role play based on the common question to make parents practice target behaviors
	Parents have reactions and are poorly prepared for joint session (A/E)	Better preparation for joint sessions	A discussion of reactions to child questions/challenges included at the end of the session
4 children	Needing an alternative to cards with emotional expressions (I)	Share pictures of facial expressions online	New supplementary material with animated facial expressions developed
3 and 5 joint	Too little time for a proper evaluation and round off (A)	Increase amount of time in the session	A separate session, session 6, added with time for evaluation and closure
	Entering the room to late and thereby miss the conversation (I)	Decide in advance when to enter the breakout room	Enter soon after initiation of the conversation
	Taking too much time before providing supervision (I)	More guidance of what to look for and when to stop	Stop immediately and provide supervision when one positive feedback and one area of improvement is identified
	Not providing supervision after listening (I)		Always provide supervision regardless of the quality of the conversation
	Long wait, or dyads leaving the session without a proper ending (A)	Give precise information about when to return to the common virtual room	Time for conversation reduced to 20 min. Provide information on when they are called back to common virtual room
Session 5	Tired or late-arriving participants in the last part of the session made evaluations hard (I)	Increase time for the session	A sixth session included involves:1) a joint evaluation of positive aspect and potential for improvements included, 2) ending of the intervention

F = Feasibility, A = Acceptability, E = Effectiveness, I=Implementation.

⁎ For both session 2 and 4.

## 4 Discussion and conclusion

### 4.1 Discussion

The current paper describes the process of developing SIBS-ONLINE, a preventive group intervention for siblings and parents of children with chronic disorders delivered via video conference. Although the thematic content and aim of the intervention are identical to the original in-person/face-to face version “SIBS”, our development process revealed that some measures had to be taken to enhance the feasibility, acceptability, potential effectiveness, and implementation of SIBS-ONLINE.

#### 4.1.1 The feasibility of online video conference interventions for siblings

The developmental process demonstrated that children were confident in use of computers and communication platforms, and that additional IT support available ahead of the intervention was not used. This is in contrast to the review findings of Banbury and colleagues [17], who concluded that training of participants may be needed. Our experiences may however be explained by the fact that our participants were children and not adults as in the review by Banbury et al. Norwegian youths have been described as experienced in use of digital technology and to show digital competence on various digital arenas [22], and previous research has described children as positive to use of technology to attain health services [18]. The fact that the developmental process overlapped with the initial Covid pandemic exacerbated both sibling and parent participants experience in interacting on communion platforms due to home schooling and home office. The opportunity provided by the pandemic for development in the field of e-health interventions has been described by several researchers in the field [18]. Despite this, we did experience some technical difficulties in Phase 1 and 3 with picture, sound, and connection problems similar to what is described in previous studies [17]. Consequently, measures were taken in the final version of SIBS-ONLINE to ensure technical support during session, such as assigning the job of providing support to the facilitator and keeping a list of contact information available during sessions.

#### 4.1.2 Acceptability of SIBS-ONLINE

In Phase 3, SIBS-ONLINE was overall described as suitable, satisfying, and attractive by the participants. The intervention was described to provide opportunities to meet likeminded and to be more available to the families as less time was spent traveling. Participating from home saved parents from the efforts and costs of needing babysitters or respite care for the child with diagnosis when participating. In line with previous studies [[16], [17], [18]] SIBS-ONLINE may, due to use of video conferencing, make a preventive intervention for a child group at risk more accessible. Another positive aspect of SIBS-ONLINE being delivered over video conference described by participants concerned privacy. For some children, sharing difficult experiences was perceived as somewhat easier when not in the same room, and that the comfort of being home made the setting more secure. This has also been described in previous research [18].

However, using the family home as the setting for delivery also had some drawbacks. Group leaders was challenged by the fact that the setting was not under their control. It is hard to eliminate possible disturbances and ensure confidentiality as you never know if anyone is nearby overhearing the conversations. However, the sibling and parent participants in Phases 1 and 3 did not express concerns regarding privacy. This is in contrast with findings of previous studies of the adult population [17] but not of studies of youth populations where concerns regarding the safety of digital health interventions have been expressed [18]. Based on reported concerns of group leaders, carefully reminding participants about confidentiality and sitting arrangements before sessions is now included in SIBS-ONLINE.

The developmental process revealed that group leaders needed to be more aware of specific communication techniques to use when conducting SIBS-ONLINE. This is, again, in line with findings from the systematic review by Banbury and colleagues [17]. However, the current developmental project adds to previous research by exemplifying these communication techniques. More specifically, group leaders needed to explicitly structure the communication in dialogue with the participants, be explicit about the schedule, their intentions, and purpose of their behavior.

The non-verbal facial communication was described as important by group leaders in Phase 1. As facial expressions were more evident on screen than it normally is in on-site groups, this provided both group leaders and facilitator to comment and involve all participants. Similarly, the non-verbal communication of the group leaders was also important. As verbal behavior such as “yes” or “mm” from the facilitator may be disrupting in online groups we suggest for example nodding, thumbs up, smiling or shaking one's head instead. Non-verbal communication with participants over video conference has previously been described difficult for clinicians [20]. Thus, we included instructions on how to use non-verbal communication in the SIBS-ONLINE manual.

Despite measures to improve communication, video conferencing somehow “locks” the opportunities of both participants and group leaders within the frame of the picture. Participants cannot look away without it easily noticed, body language is invisible, and the silence when nobody talks may feel more oppressive. Thus, to increase acceptability of SIBS-ONLINE we suggest aiming for at least five participants to put each participant less “in the spotlight” and increase the total amount of verbal participation.

Previous researchers have explained the lower satisfaction with interventions over video conference among clinicians compared to patients with their work becoming more complex due to having to adapt communication [20]. When developing the SIBS-ONLINE intervention this challenge has been met by including specific sections in the SIBS-ONLINE manual on how to adapt the communication to the video conference mode and we also suggest practicing these communication skills before running SIBS-ONLINE groups.

#### 4.1.3 Potential effectiveness of SIBS-ONLINE

The original in-person version of SIBS has shown promising results when it comes to strengthening family communication, increasing sibling knowledge about the diagnosis and reducing psychological problems [13]. Currently we cannot draw any conclusions about the effectiveness of SIBS-ONLINE based on the developmental work presented in this paper. However, in line with Banbury's definition of effectiveness [17] we recorded that participants in SIBS-ONLINE reported to learn communication techniques, perceived have important conversations during the intervention, share important experiences, and feel support in the groups. They also gave feedback on how SIBS-ONLINE may become more effective e.g. by increasing the number of sessions, clearer communicate information, and conduct group on a time of the day where children were less tired. This was incorporated in the final SIBS-ONLINE and the effectiveness of the intervention will be the focus of our future research.

#### 4.1.4 Is SIBS-ONLINE a reliable video conference version of SIBS?

Although previous research describes high reliability and validity of interventions when delivered over video conference [17] we found some of the material and tasks from the original SIBS hard to implement over video conference. Thus, this developmental process resulted in development of SIBS-ONLINE material adapted to the delivery mode. For example, in the original SIBS, group leaders use flipchart to draw, and take notes to underline and exemplify what has been shared in the group. In SIBS-ONLINE, the use of computer programs and web meeting applications are suggested to share drawings and notes with the group in a similar manner. However, the use of e.g. a drawing program might compromise the quality of the session as sharing a document on the screen reduces the size of the pictures of the participants and make non-verbal communication harder to read, and use of unfamiliar programs may also demand to much attention from the group leaders.

Screen fatigue among participants in educational sessions over video conference has previously been described [21]. Use of short movies during parent sessions in SIBS-ONLINE was greatly appreciated by the group leaders as it makes the sessions varied and may lessen screen fatigue among participants. The pilot-testing however, made it evident that new, shorter, and more specific psychoeducational videos could improve the sessions even further, and that these should be made available for parents between sessions and after SIBS-ONLINE.

In the developmental process group leaders did experience some challenges in relation to the youngest participants (<12 years) ability to take part in the sessions over video conference. This may also be related to screen fatigue, more specifically, younger children's ability to sit still and keep focused during longer verbal group discussions over video conference. Although measures such as reminding the children about the need of limiting sounds and movement were suggested, future research should aim to establish the age groups best suited for SIBS-ONLINE and explore measures to better adapt the intervention for younger children.

#### 4.1.5 Limitations

The developmental process described in this report has limitations that warrant attention. One limitation is that the participants in Phase 1 and 3 do not necessarily represent the population of families/siblings of children with chronic disorders. The participants self-referred to the groups based on needs and interest. As a consequence, the feedback we based the development of SIBS-ONLINE on may only be reflecting a selected group. Second and relatedly, most of the participants were girls so we need more information on the perceived acceptability of boys as participants. Third, the group leaders in Phase 1 were experienced with using communication platforms to offer services to families of children with chronic disorders. Thus, some difficulties and aspects worth mentioning in the SIBS-ONLINE manual might have gone unnoticed. Finally, the deliverer of SIBS-ONLINE in Phase 3 was a national resource center and might have other facilities and resources available to deliver video conference groups than a municipality has. A study of implementation of SIBS-ONLINE in a municipal setting is thus needed.

### 4.2 Innovation

To our best knowledge the developmental process described in this report resulted in the first video conference intervention to prevent psychological difficulties in siblings of children with chronic health disorders. This answers to a psychological need identified in the target population and in countries with low density population areas. The Covid pandemic shed light upon an increasing need for online interventions. The work presented in this paper may provide inspiration, examples and advice for developers and deliverers of such interventions in the future.

### 4.3 Conclusion

The result of the developmental process was the SIBS-ONLINE intervention, and corresponding intervention material addressing the important aspects learned in the developmental process. Although the topics, aims and methods are the same, the delivery mode is different, new material necessary, and communication aspects experienced as somewhat different and complicated. To establish whether SIBS-ONLINE has the similar outcomes as the original in-person version of SIBS upon family communication, sibling psychological adaption and illness knowledge, research must be conducted.

We summarize our experiences with conducting video conference groups in the following list of advice for clinicians:

• Five to seven participants per group seems to be an optimal group size.
• Choose communication platform based on needs for functions ensuring optimal pictures of participants.
• Practice using the technical solutions before leading groups.
• Have a back-up plan for technical problems and technical assistance if possible.
• Practice and use specific verbal communication techniques during video conference groups.
• Be aware of and use non-verbal communication deliberately.
• Vary the content and form of sessions to keep participants' attention.

## Declaration of Competing Interest

The authors declare that they have no known competing financial interests or personal relationships that could have appeared to influence the work reported in this paper.
